# Supplementary material for: High Adherence to the Mediterranean Dietary Pattern Is Inversely Associated with Systemic Inflammation in Older but Not in Younger Brazilian Adults
Source: Nutrients. 2024 May 2;16(9):1385. doi: 10.3390/nu16091385 (PMC11085370; doi:10.3390/nu16091385)
Supplement: Supplementary file 1 [file nutrients-16-01385-s001.zip › nutrients-2746697-supplementary.pdf]

Supplementary file

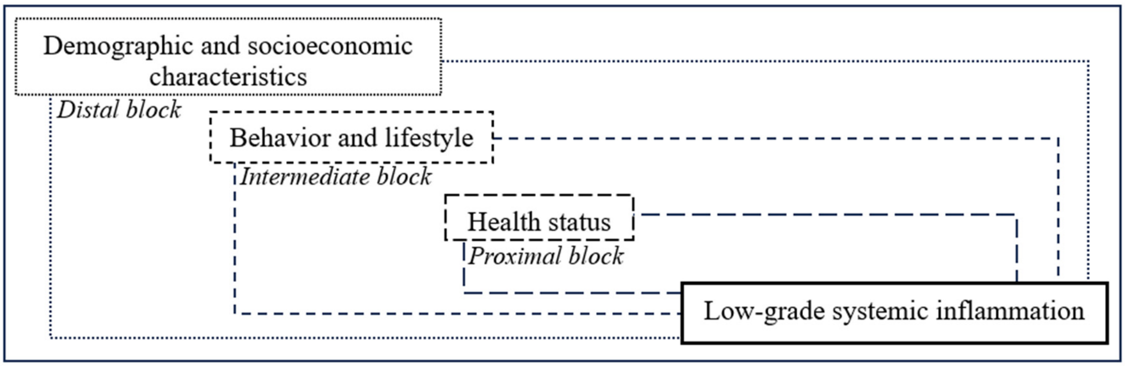

Figure S1. Hierarchical model for analyzing factors associated with low-grade systemic inflammation.
